# Ultrasound-guided pulsed radiofrequency neuromodulation of the suprascapular nerve in partial rotator cuff tears

**DOI:** 10.3906/sag-1906-132

**Published:** 2019-10-24

**Authors:** Ender SİR, Sami EKSERT

**Affiliations:** 1 Department of Pain Medicine, Health Sciences University, Gülhane Training and Research Hospital, Ankara Turkey; 2 Department of Anesthesia and Reanimation, Health Sciences University, Gülhane Training and Research Hospital, Ankara Turkey

**Keywords:** Pulsed radiofrequency treatment, rotator cuff injuries, ultrasonography, nerve block, shoulder pain

## Abstract

**Background/Aim:**

Pulsed radiofrequency (PRF) of the suprascapular nerve has been shown to be effective in the treatment of chronic shoulder pain. Ultrasound (US) guidance has gained popularity in regional blocks recently. This study aims to investigate the efficacy of suprascapular nerve pulsed radiofrequency under the guidance of ultrasonography.

**Materials and methods:**

This retrospective study included patients treated with PRF of the suprascapular nerve with a diagnosis of partial rotator cuff tears. The patients were assessed with a numeric rating scale (NRS), the Shoulder Pain and Disability Index (SPADI), and a Likert patient satisfaction score before the treatment and 3 weeks and 6 months following the treatment.

**Results:**

A total of 31 patients was included in the study. The patients’ mean age was 66.8 ± 13.3 years. The mean scores of the NRS, SPADI, and Likert scale before the procedure (7.32 ± 1.1, 69.0 ± 8.5, 1.6 ± 0.6) and at 3 weeks (2.9 ± 2.1, 32.1 ± 17.20, 4 ± 1.2) and 6 months (3.2 ± 2.6, 33.9 ± 20.8, 3.8 ± 1.2) after the procedure were evaluated. We observed significant improvement in NRS, SPADI, and Likert scores at 3 weeks and 6 months following the treatment (P < 0.001).

**Conclusion:**

The study demonstrated that US-guided suprascapular nerve PRF achieves good pain relief and functional improvement in patients with partial rotator cuff tears for at least 6 months.

## 1. Introduction

Management of shoulder pain requires a multimodal and algorithmic approach, including the use of nonsteroidal antiinflammatory drugs (NSAIDs), physiotherapy, selective nerve interventions, and surgical procedures [1]. Generally, a suprascapular nerve block is administered first with local anesthetic agents and corticosteroids [2]. The technique is often useful only for the short term, and repeated interventions are needed. Thus, the risk of nerve injury, infection, and side effects due to steroid use may increase [3]. Other therapeutic options, including neurolysis or neurectomy of the suprascapular nerve, may cause permanent paralysis of the supraspinatus and infraspinatus muscles [4].

Suprascapular nerve pulsed radiofrequency (PRF) neuromodulation has emerged as an alternative intervention for pain control since 2002 and has been increasingly used to date [5]. Recent studies have reported that suprascapular nerve PRF under ultrasound (US) guidance provides direct visualization of the nerve, thereby allowing the more rapid onset of anesthesia [6,7]. The main advantage of US-guided suprascapular nerve PRF over other pain management methods is that a single application provides long-term pain relief with a lower incidence of neural trauma [4,8,9]. However, there are limited studies evaluating the usefulness of the procedure in partial rotator cuff tears under US guidance [10].

In this study, we aimed to investigate the efficacy of USguided suprascapular nerve PRF on chronic shoulder pain and function in patients with partial rotator cuff lesions.

## 2. Materials and method

### 2.1. Study design and study population

This retrospective study included 31 patients (24 women, 7 men) who underwent US-guided suprascapular nerve PRF between May 2016 and November 2018 and who had shoulder pain for at least 3 months due to partial rotator cuff tear. Written informed consent was obtained from each patient. Patients’ data were obtained from patient files and follow-up forms. The institutional review board approved the study protocol (2019/06, 19/71), and the study was conducted according to the principles of the Declaration of Helsinki.

The inclusion criteria for the study were as follows: refractory shoulder pain unresponsive to conservative therapies including paracetamol, NSAIDs, opioids, physiotherapy, intraarticular steroid injections, or combinations of these treatments, and radiologically proven partial tear of the rotator cuff. Exclusion criteria included inflammatory arthritis, adhesive capsulitis, active synovitis of the shoulder joint, previous history of shoulder surgery, shoulder joint injection in the last 1 month, advanced osteoarthritis, neurologic conditions (hemiparesis, Parkinson’s disease, etc.), current use of anticoagulant medications, and presence of complete tear of the rotator cuff. All patients underwent shoulder radiography before the treatment, and the etiology of a partial tear of the rotator cuff was documented by magnetic resonance imaging (MRI) findings. A radiologist evaluated the MRIs of the patients. MRI revealed muscle atrophy in 6 (19%) patients; nevertheless, the volume of muscle was larger than that of fat (muscle > fat). On the other hand, average muscle volume was observed in the remaining 25 (81%) patients. Humeral head migration and cysts were not observed in any patient. Patients were evaluated using a numeric rating scale (NRS) for pain, ranging from none (0) to extreme (10). The Shoulder Pain and Disability Index (SPADI), a 13-item scale, was used to assess improvement in shoulder function [11]. A 5-point Likert scale, a subjective assessment method, was used to evaluate patient satisfaction. The NRS, SPADI, and Likert measurements were performed before the treatment and at 3 weeks and 6 months after the treatment.

### 2.2. Intervention

**Figure 1:**
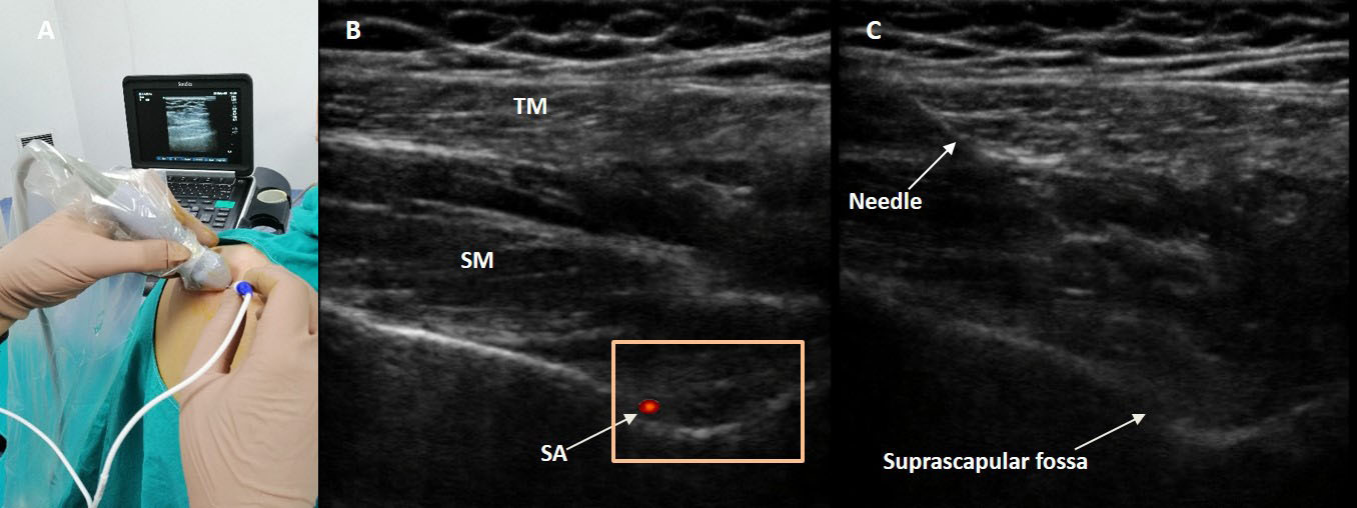
A) The positioning of the linear ultrasound transducer and radiofrequency electrode. B) Scanning of the suprascapular nerve with linear ultrasound probe; trapezius muscle (TM), suprascapular muscle (SM), suprascapular notch, and color Doppler imaging of the suprascapular artery (SA). C) Real-time imaging of the needle insertion under ultrasonographic guidance.

Two physicians experienced in US-guided suprascapular nerve injections performed the PRF procedures under local anesthesia in an operating room. After the patient was placed in a sitting position, intravenous access was established and routine monitoring (pulse oximetry, electrocardiogram, and noninvasive arterial pressure) was performed. Mild sedation was achieved with 2 mg of intravenous midazolam bolus at a dose that did not impair the patient’s consciousness. Chlorhexidine was used for skin antisepsis. The suprascapular notch and the advance of the needle into the suprascapular nerve were visualized by US (Edge, Sonosite, Bothell, WA, USA) with a highfrequency linear probe (HFL50xp, 15-6 MHz) (Figures 1A and 1B). Skin anesthesia was achieved by administering 2% prilocaine through a 25-G needle. For an in-plane approach, a 22-G, 10-cm-long echogenic radiofrequency (RF) cannula with 5-mm active tip (EchoRF, Cosman, Burlington, MA, USA) was introduced to the suprascapular notch (Figure 1C). Motor stimulation was performed with 2 Hz at a setting of 1 V, and the response was observed at the deltoid muscle. Subsequently, sensory stimulation was performed at 50 Hz at a setting of 0.5 V. Patients defined paresthesia, tingling, and pain in the deltoid and upper arm region. Accurate placement of the needle tip was demonstrated via US. After negative aspiration of blood, 1 mL of 1% prilocaine was injected. One minute after local anesthetic injection, pulsed RF was performed at 42 °C for 360 s. Patients were followed in the postoperative care unit for 1 h as postprocedural complications could develop.

### 2.3. Statistical analysis

After the data were transferred to a computer, statistical analyses were performed using SPSS 21.0 (IBM Corp., Armonk, NY, USA). Descriptive statistics were defined as number, percentage, mean, standard deviation (SD), minimum, and maximum values. The consistency of continuous data with normal distribution was determined by the Kolmogorov–Smirnov test. The Friedman test was used to compare continuous data in dependent triple groups that did not conform to normal distribution. The Bonferroni corrected Wilcoxon test was used to determine which binary subgroup was the origin of the difference in the triple groups. In Bonferroni correction, statistical significance level was accepted as P ˂ 0.017. In other tests, P ˂ 0.05 was considered significant.

## 3. Results

**Figure 2:**
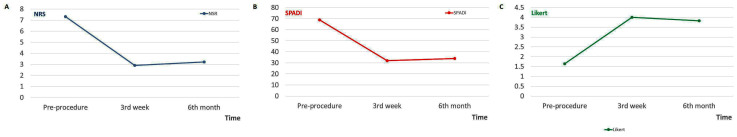
Diagram of numeric rating scale (NRS) (A), Shoulder Pain and Disability Index (SPADI) (B), and Likert patient satisfaction (C) scores before and after pulsed radiofrequency therapy.

The present study included 31 shoulders of 31 patients who underwent US-guided PRF procedures of the suprascapular nerve. The mean age of the patients was 66.8 ± 13.3 years, and the mean body mass index (BMI) was 28.1 ± 2.7. The demographic and clinical characteristics of the patients included in the study are presented in Table 1. Significant improvements in NRS and SPADI subscores were observed in the treated patients in the third week and sixth month after the procedure when compared to the preprocedural scores (P < 0.001) (Table 2). In addition, no statistically significant difference was observed between NRS and SPADI scores at 3 weeks and at 6 months (P = 0.28, P = 0.44). Based on the results of the Likert scale, suprascapular nerve PRF treatment resulted in good patient satisfaction in 71% patients (22 patients out of 31) at 3 weeks (P < 0.001) and in 68% patients (21 out of 31) at 6 months (P < 0.001) (Table 2; Figure 2). No adverse effects or complications were observed throughout the follow-up period of 6 months.

**Table 1:** Demographic data.

	n: 31
SexMaleFemale	7 (22.6)24 (77.4)
Age (years)	66.77 ± 13.29
Height (cm)	163.00 ± 6.85
Weight (kg)	74.64 ± 7.56
BMI	28.12 ± 13.29
Side (left/right)	18/13

**Table 2:** Numeric rating scale (NRS), Shoulder Pain and Disability Index (SPADI), and Likert patient satisfaction scores before treatment and 3 weeks and 6 months after treatment

		Mean ± std. deviation	P
NRS	Before treatment	7.32 ± 1.10	<0.001
	3rd week	2.90 ± 2.11	
	Before treatment	7.32 ± 1.10	<0.001
	6th month	3.22 ± 2.61	
	3rd week	2.90 ± 2.11	0.28
	6th month	3.22 ± 2.61	
SPADI	Before treatment	68.96 ± 8.54	<0.001
	3rd week	32.09 ± 17.20	
	Before treatment	68.96 ± 8.54	<0.001
	6th month	33.93 ± 20.78	
	3rd week	32.09 ± 17.20	0.44
	6th month	33.93 ± 20.78	
Likert	Before treatment	1.64 ± 0.60	<0.001
	3rd week	4.00 ± 1.15	
	Before treatment	1.64 ± 0.60	<0.001
	6th month	3.83 ± 1.15	
	3rd week	4.00 ± 1.15	0.09
	6th month	3.83 ± 1.15	

## 4. Discussion

In this study, the efficacy of US-guided suprascapular nerve PRF treatment on chronic shoulder pain related to partial rotator cuff tears was investigated. During the 6-month follow-up period, most patients demonstrated good pain relief and improved shoulder functionality. To our knowledge, this is one of the few studies investigating the use of the US in the application of PRF to the suprascapular nerve.

Along with the motor innervation of the infraspinatus and supraspinatus muscles, the suprascapular nerve covers approximately 70% of the sensory innervation of the shoulder girdle, including the glenohumeral joint, capsule, and acromioclavicular joint [12]. Correspondingly, an isolated blockade of the suprascapular nerve has been demonstrated to be effective in pain relief after shoulder surgeries [13]. Suprascapular nerve block has been performed in joint pathologies, rotator cuff lesions, and other related conditions, providing effective pain relief and functional improvement [13–16]. The use of PRF on peripheral nerves such as the suprascapular nerve has gained popularity in recent years owing to the nondestructive mechanism and low risk of complications. Although the mechanism of pain relief of PRF is not clearly understood, it has been proposed that an electrical field is generated at the tip of the needle that penetrates the nerve fibers and causes physiological and ultrastructural changes in the nociceptive axons [17]. Another proposed mechanism is that an increase in c-Fos production occurs in the posterior horn cells after PRF, possibly affecting the C-fiber transmission by altering the activity of the sodium channels [18,19]. Thus, longer duration of pain relief can be achieved with this technique compared to other treatment modalities such as injections of corticosteroids and local anesthetic agents or thermal lesioning [10].

In the literature, the first application of PRF to the suprascapular nerve was applied by Rohof in 2002 with a blind technique [5]. Although the blind technique is still widely used, it may cause catastrophic complications such as pneumothorax, especially in patients with anatomical variations [20]. However, Gurbet et al. reported significant pain relief and increase in shoulder function for at least 3 months after the blind technique suprascapular PRF procedure without any severe complications [21]. Instead of the blind technique, fluoroscopy or computed tomography (CT)-guided techniques have been applied in PRF of the suprascapular nerve [22]. However, in USguided procedures, needle advancement is displayed in real time, thereby reducing the likelihood of damage to nerves, vessels, and other adjacent structures [23– 25]. Furthermore, when US is compared with CT and fluoroscopy, US does not cause radiation exposure to the patient or researcher, it is a portable device, and it reduces the cost of the procedure [26]. Correspondingly, a trend towards US use has been observed in recent studies. Wu et al. reported improved shoulder function and pain relief for at least 12 weeks after the US-guided suprascapular nerve PRF procedure for adhesive capsulitis and concluded that a noticeable reduction in VAS scores could be achieved as early as 1 week after the procedure [14]. In a recent study, Ergonenc and Beyaz performed US-guided suprascapular PRF for 74 patients and achieved significant improvements in pain and functionality in the majority of patients during the 6-month follow-up period [8]. Therefore, US guidance was the preferred technique instead of fluoroscopy or CT guidance in this study. As a result, US-guided suprascapular nerve PRF showed significant improvement in shoulder pain and function through the 6-month follow-up period.

There were some limitations to our study. The study lacked a control group, and comparison of US-guided suprascapular nerve PRF with other treatment modalities was not possible. Although good results were obtained at the end of the 6-month follow-up period, further studies are needed to evaluate the long-term effects of US use on PRF application to the suprascapular nerve. Finally, due to the rigid inclusion criteria, the number of patients included in the current study was relatively small, and this may limit the generalizability of the results of the study.

In conclusion, the current study demonstrated that US-guided suprascapular nerve PRF is a reliable technique in partial rotator cuff tears. In the majority of patients, it provides adequate pain relief and an improvement in shoulder functions for at least 6 months. Furthermore, trained physicians can easily repeat this neuromodulation procedure in the case of recurrence of pain without any damage to the nerve and neighboring soft tissues under US guidance.
